# The Role of Aquaporin and Tight Junction Proteins in the Regulation of Water Movement in Larval Zebrafish (*Danio rerio*)

**DOI:** 10.1371/journal.pone.0070764

**Published:** 2013-08-14

**Authors:** Raymond W. M. Kwong, Yusuke Kumai, Steve F. Perry

**Affiliations:** Department of Biology, University of Ottawa, Ottawa, Ontario, Canada; Biological Research Centre of the Hungarian Academy of Sciences, Hungary

## Abstract

Teleost fish living in freshwater are challenged by passive water influx; however the molecular mechanisms regulating water influx in fish are not well understood. The potential involvement of aquaporins (AQP) and epithelial tight junction proteins in the regulation of transcellular and paracellular water movement was investigated in larval zebrafish (*Danio rerio*). We observed that the half-time for saturation of water influx (*K*_u_) was 4.3±0.9 min, and reached equilibrium at approximately 30 min. These findings suggest a high turnover rate of water between the fish and the environment. Water influx was reduced by the putative AQP inhibitor phloretin (100 or 500 μM). Immunohistochemistry and confocal microscopy revealed that AQP1a1 protein was expressed in cells on the yolk sac epithelium. A substantial number of these AQP1a1-positive cells were identified as ionocytes, either H^+^-ATPase-rich cells or Na^+^/K^+^-ATPase-rich cells. AQP1a1 appeared to be expressed predominantly on the basolateral membranes of ionocytes, suggesting its potential involvement in regulating ionocyte volume and/or water flux into the circulation. Additionally, translational gene knockdown of AQP1a1 protein reduced water influx by approximately 30%, further indicating a role for AQP1a1 in facilitating transcellular water uptake. On the other hand, incubation with the Ca^2+^-chelator EDTA or knockdown of the epithelial tight junction protein claudin-b significantly increased water influx. These findings indicate that the epithelial tight junctions normally act to restrict paracellular water influx. Together, the results of the present study provide direct *in vivo* evidence that water movement can occur through transcellular routes (via AQP); the paracellular routes may become significant when the paracellular permeability is increased.

## Introduction

Although maintaining water balance is fundamental to the physiology of teleost fish [1]–[3], the molecular mechanisms regulating water movement across the gill/skin remain poorly understood. Previous studies in mammals [4] and fish [5] have suggested that epithelial water flux occurs, at least in part, through transcellular pathways formed via aquaporins (AQPs). To date, 13 and 18 different AQPs have been identified in mammals [6] and fish [7], respectively. AQP1 has an important physiological role in promoting water reabsorption in the mammalian proximal tubule [8]. Thus, knockout of AQP1 in mice was shown to reduce water reabsorption from the renal tubules, thereby increasing the production of dilute urine and ultimately causing dehydration [9], [10].

The orthologs of AQP1 have been identified in several teleost species including the European eel *Anguilla anguilla* [11], black seabass *Centropristis striata* [12], rainbow trout *Oncorhynchus mykiss* [13], Atlantic salmon *Salmo salar* [14] and zebrafish *Danio rerio* [7]. Giffard-Mena et al. [15] reported that the AQP1 mRNA expression in the kidney of seabass (*Dicentrarchus labrax*) was higher in seawater (SW)- than in freshwater (FW)-acclimated fish. Similarly, Tipsmark et al. [14] showed that the AQP1a mRNA levels were increased in the kidney of Atlantic salmon following SW-acclimation. These findings suggest that during acclimation to a hyperosmotic environment, increasing renal AQP1a expression may play a role in enhancing water reabsorption by the kidney.

In adult zebrafish, AQP1a is reported to be ubiquitously expressed [7], [16], while AQP1b is expressed only in the ovary, testis and brain [7]. In larval zebrafish, the results of *in situ* hybridization demonstrated that AQP1a is expressed on the skin of the yolk sac, presumably to regulate water influx [16], [17]. However, it is still unclear whether AQP1 has any physiological role in facilitating transcellular water movement in teleost fish *in vivo*. It is well documented that the presumptive AQP inhibitors, phloretin [18]–[20] and acetazolamide [21], [22], can reduce water influx into *Xenopus* oocytes or cell lines expressing the mammalian AQPs. To our knowledge, their effects on water flux have not been studied in fish.

FW teleosts are hyperosmotic to their environment and thus maintaining a tight epithelium is important to prevent excessive water influx via paracellular routes. Paracellular properties are governed by tight junctions (TJs), which are composed of several different classes of transmembrane proteins, including occludin and members of the claudin family. Claudins can form either paracellular barriers to limit diffusion, or channels to assist diffusion depending on their molecular properties [23]. We have previously reported that translational gene knockdown of a specific tight junction protein, claudin-b, in larval zebrafish increases the flux of the paracellular permeability marker polyethylene glycol (PEG-4000) by about 15% [24], [25]. Additionally, a portion of the larvae deficient in claudin-b exhibited fluid accumulation in the pericardial and yolk sac [24], presumably owing to an increase in paracellular water influx. In adult FW teleosts, water flux occurs primarily at the gills and can account for as much as 90% of the total body water influx [3]. However, developing zebrafish do not possess a functional gill until at least 7 days post fertilization (dpf) [26], and therefore water influx prior to this developmental stage presumably occurs either through drinking and/or diffusion across the body surface. However, the relative contributions of drinking and transcellular or paracellular fluxes to overall water uptake in larval zebrafish have not been evaluated.

With the above background, we hypothesized that water movement in larval zebrafish occurs transcellularly through the specific involvement of AQP1a1 water channels and that TJs play a critical role in restricting paracellular water influx. The objectives of the present study were three-fold; i) to assess the contribution of drinking to water uptake, ii) to evaluate the involvement of AQPs in facilitating transcellular water movement, and iii) to determine whether tight junctions serve to provide a barrier to water flux.

## Materials and Methods

### Ethics Statement

The experiments were conducted in compliance with guidelines of the Canadian Council of Animal Care and after the approval of the University of Ottawa Animal Care Committee (Protocol BL-226).

### Fish

Adult zebrafish (*Danio rerio* Hamilton-Buchanan 1822) were purchased from Big Al's Aquarium Services (Ottawa, ON, Canada) and kept in the University of Ottawa Aquatic Care Facility. The fish were maintained in plastic tanks supplied with aerated, dechloraminated City of Ottawa tap water at 28°C. The ionic composition of the water was (in mM) Na^+^  = 0.78; Cl^−^  = 0.4; Ca^2+^  = 0.25; K^+^  = 0.025; pH 7.6. Fish were subjected to a constant 14∶10-h light-dark photoperiod and fed daily until satiation with No. 1 crumble-Zeigler (Aquatic Habitats, Apopka, FL). Morpholino and sham injected embryos (details below) were reared in 50 ml Petri dishes supplemented with dechloraminated City of Ottawa tap water (pH 7.6). The Petri dishes were kept in incubators set at 28.5°C. All analyses were performed at 4 dpf except where mentioned otherwise. The dry weight for each larva at 4 dpf was approximately 0.6 mg.

### Measurements of water influx

The influx of water was measured using a radiotracer method. To evaluate the time-course of water influx, separate groups of fish were exposed to 1 µCi/ml of ^3^H_2_O (American Radiolabeled Chemicals Inc., USA) for 2, 5, 10, 15, 30, 45 or 60 min. At the end of the flux period, fish were killed with an overdose of tricaine methanesulfonate (MS-222), briefly washed in isotope-free water, and three larvae were pooled as one sample (n = 1). Water samples were also collected to determine ^3^H_2_O specific activity. Fish were digested with a tissue solubilizer (Solvable^TM^; Perkin Elmer, USA) and later neutralized using glacial acetic acid. Subsequently, the radioactivity in the digest as well as the water samples was measured using a liquid scintillation counter (LS-6500; Beckman Coulter Co., Canada) following addition of scintillation cocktail (BioSafe-II; RPI co. Mt. Prospect, IL, USA). Because we observed that the half-time for saturation of water influx was approximately 5 min (see results), a 5-min flux period was conducted for all subsequent experiments.

### Measurements of drinking

To examine the contribution of drinking to overall water influx, drinking rates were measured as described previously [27], [28]. Fish were exposed to 2 μCi/ml of 70 kDa [^14^C]dextran (American Radiolabeled Chemicals Inc., USA). Following 0.5, 1 and 1.5 h of exposure, fish were killed with an overdose of MS-222, briefly rinsed in isotope-free water, and radioactivity was measured as detailed above. Using autoradiography, it was previously shown that the absorption of 70 kDa dextran is confined only to the gastrointestinal lumen in the larval cod *Gadus morhua* [28]. Therefore, the accumulation of 70 kDa dextran estimates the maximal amount of water that can be absorbed by drinking.

### Pharmacological experiments

The effects of putative AQP blockers phloretin and acetazolamide (Sigma-Aldrich, USA) on water influx were examined. Fish were first incubated with phloretin (100 or 500 µM) or acetazolamide (200 µM) for 15 min, and measurement of water influx was performed as described above. The concentrations of phloretin and acetazolamide were chosen based on previous studies of water transport in mammals [21], [29]. Although mercury (Hg^2+^) was reported to be an effective AQP inhibitor *in vitro* [5], [7], in the present study we observed that mercury was highly toxic to larval zebrafish, which prevented its further use (see discussion).

### Western blot analysis

Methods for western blot analysis were similar to those reported previously [30]. Briefly, twenty larvae were pooled as one sample (n = 1) and homogenized in a RIPA buffer (150 mM NaCl, 1% Triton X-100, 0.5% sodium deoxycholate, 0.1% SDS, 50 mM Tris-HCl, 1 mM EDTA, 1 mM phenylmethanesulfonyl fluoride) plus protease inhibitor cocktail (Roche, USA). For western blotting of AQP1a1, extracted protein was heated for 10 min at 70^o^C, loaded onto a 10% SDS-PAGE and transferred to a PVDF membrane (Bio-Rad, USA). The membrane was blocked for 1 h with 5% skimmed milk, probed with eel anti-AQP1a (VNGPDDVPAVEMSSK; 87% identical to the zebrafish AQP1a at the C-terminal) at 1∶1000 dilution in 2% skimmed milk, and then incubated at 4^o^C overnight. Subsequently, the membrane was probed with 1∶15,000 goat anti-rabbit secondary antibody (Pierce, USA) for 2 h at room temperature, and the immunoreactive bands were detected using Luminata Western HRP Substrates (Millipore, USA). Methods for western blotting with claudin-b antibody have been described elsewhere [30]. To check for equal protein loading, the membrane was re-probed with *β*-actin antibodies (1∶4000; A2066, Sigma-Aldrich) for 2 h at room temperature after stripping with Re-Blot Plus solution (Millipore, USA). The intensity of the bands was estimated using ImageJ (Rasband 2006, http://imagej.nih.gov/ij/), and the protein expression was normalized to that of β-actin.

### Immunohistochemistry

To examine the localization of AQP1a1 and its possible expression in ionocytes, 4 dpf larvae were incubated with 50 µg/L of Alexa-633 conjugated concanavalin-A (ConA) and Alexa-596 conjugated Mitotracker® (Invitrogen, Burlington, ON, Canada) for 30 min. ConA and Mitotracker® are known to label H^+^-ATPase-rich cells and mitochondrion-rich cells, respectively. After incubation, the fish were washed briefly in PBS and fixed overnight in 4% paraformaldehyde in PBS at 4^o^C. Following fixation, the fish were washed with PBST (PBS plus 0.1% Tween) and incubated overnight with the AQP1a1 antibody (1∶200 dilution) in PBST containing 3% goat serum, 1% BSA and 0.4% Triton-X at 4^o^C. Subsequently, the fish were incubated in the dark with an Alexa 488-conjugated goat anti-rabbit IgG (Invitrogen; 1∶500 dilution) for 2 h at room temperature. The images were acquired using a Zeiss LSM 5 Pascal/AxioVert 200 confocal microscope (Carl Zeiss; Jena, Germany).

To examine whether AQP1a1 was also expressed in Na^+^/K^+^-ATPase-rich cells (NaR), 4 dpf fish were stained with both AQP1a1 and Na^+^/K^+^-ATPase antibody (α5, diluted 1∶300 in PBST; Developmental Studies Hybridoma Bank, University of Iowa) after fixation. The AQP1a1 and Na^+^/K^+^-ATPase were then labeled with rabbit Alexa 488- and mouse Alexa 596-conjugated secondary antibody, respectively, and images were acquired as described above. To determine whether AQP1a1 was expressed at the apical or basolateral membrane, immunostaining of AQP1a1 with ConA or Na^+^/K^+^-ATPase was performed (ConA and Na^+^/K^+^-ATPase stain the apical and basolateral membranes, respectively). *Z*-stack images were then obtained using confocal microscopy.

### Morpholino knockdown of AQP1a1 protein expression

To evaluate the potential involvement of AQP1a1 in transcellular water influx, a morpholino oligonucleotide (5′-AAG CCT TGC TCT TCA GCT CGT TCA T-3′; Genetools, OR, USA) was designed to block the translation of zebrafish AQP1a1 (GenBank ID: NM 207059.1). The morpholino was diluted in a Danieau buffer [58 mM NaCl, 0.7 mM KCl, 0.4 mM MgSO_4_, 0.6 mM Ca(NO_3_)_2_, 5.0 mM HEPES (pH 7.6)] plus 0.05% phenol red before injection. A “sham” group was injected with a standard control morpholino (5′-CCT CTT ACC TCA GTT ACA ATT TAT A-3′; GeneTools) prepared as the AQP1a1 morpholino. 4 ng of morpholino (1 nl) was injected into one-cell stage embryos using a microinjector system (model IM 300; Narishige, Long Island, NY). No gross morphological abnormalities were observed following AQP1a1 morpholino knockdown.

### Examination of the role of tight junctions in paracellular water influx

The potential involvement of tight junctions in reducing paracellular water influx was examined. Fish were incubated with 1 mM sodium ethylenediaminetetraacetate (EDTA) in Ca^2+^-free water for 30 min, and measurement of water influx was conducted as detailed above. EDTA is a Ca^2+^-chelator and is known to disrupt the integrity of TJs [31]. Controls were also performed in the presence and absence of Ca^2+^. The Ca^2+^-free water was prepared with double-deionized water supplemented with NaCl, MgSO_4_·7H_2_O, K_2_HPO_4_ and KH_2_PO_4_, pH adjusted to 7.6 with the addition of 1 N NaOH. The control water was prepared in a similar fashion with the addition of CaSO_4_·2H_2_O. The final concentrations of Na^+^, Ca^2+^, Mg^2+^ and K^+^ in the control water were (in mM) 0.78, 0.25, 0.15 and 0.3, respectively.

The role of tight junction protein in paracellular water influx was examined further using a gene knockdown approach. The expression of claudin-b (GenBank ID: NM131763.2), which is expressed on the larval skin, was knocked down by morpholino injection (2 ng; 5′- CCG GTT GAT GCC ATG CTT TTT CGT T -3′; Genetools, OR, USA) as described above. Because we observed that the epithelial permeability to PEG was significantly increased in 3 dpf claudin-b morphants [24], water influx was measured at this developmental stage. The effectiveness of this morpholino in preventing claudin-b expression was reported previously [24].

### Real-time PCR

The effects of AQP1a1 knockdown on claudin-b mRNA expression, and of claudin-b knockdown on AQP1a1 expression, were evaluated. Methods for RNA extraction, cDNA synthesis and real-time PCR analysis were performed as described previously [25]. All RT-qPCR assays were performed on a real-time MX3000P qPCR system using Brilliant III SYBR Green Master Mix (Agilent Technologies, USA). Specific primers were used to amplify the transcripts of AQP1a1 (forward: 5′-GCG ACT GTG GCC AGC GCT AT-3′; reverse: 5′-CGT CCC GCC GCC TTT TGT CT-3′) and claudin-b (forward: 5′-AGA CAG CGG AAA ATA CAC AGC-3′; reverse: 5′-TGA GCC TCA ATG TCC AAC AA-3′). The expression of a housekeeping gene 18S was also assessed. All RT-qPCR was performed using the following conditions: 95°C for 3 min, 40 cycles of 95°C for 20 s and 58°C for 20 s, with final extension for 5 min at 72°C. All data were normalised to the expression of 18S, and the relative mRNA expression was calculated based on the method described by Pfaffl [32].

### Calculations and statistical analysis

The time course of water influx was evaluated using non-linear regression analyses:
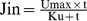
where “U_max_” represents the steady state accumulation (equilibrium) of water, “K_u_” represents the half-time saturation of water influx, and t is the duration of influx (min).

Influx of ^3^H_2_O was calculated by:
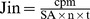
where ‘cpm’ represents counts per minute measured in the fish, ‘SA’ is the specific activity in the water, “n” is the number of fish, and ‘t’ is the duration of the experiment (in minutes for ^3^H_2_O influx. Drinking rate was calculated as the slope of a regression line passing through the origin with total incorporated radioactivity of 70kDa [^14^C]dextran as the dependent variable and flux period (h) as the independent variable.

Statistical analyses were performed using Sigmaplot^®^ (version 11.2; SystatSoftware, Inc., USA). All data were analyzed either by Student's *t*-test or one-way analysis of variance followed by a post-hoc Holm-Sidak test. For the phloretin experiment with AQP1a1 morphants, a two-way ANOVA was performed (morpholino knockdown and phloretin treatment were treated as two independent variables). Data are reported as the mean ± SEM; *p*<0.05 was taken as the level of significance.

## Results

### Time-course of water influx and contribution of water drinking

**Figure 1:**
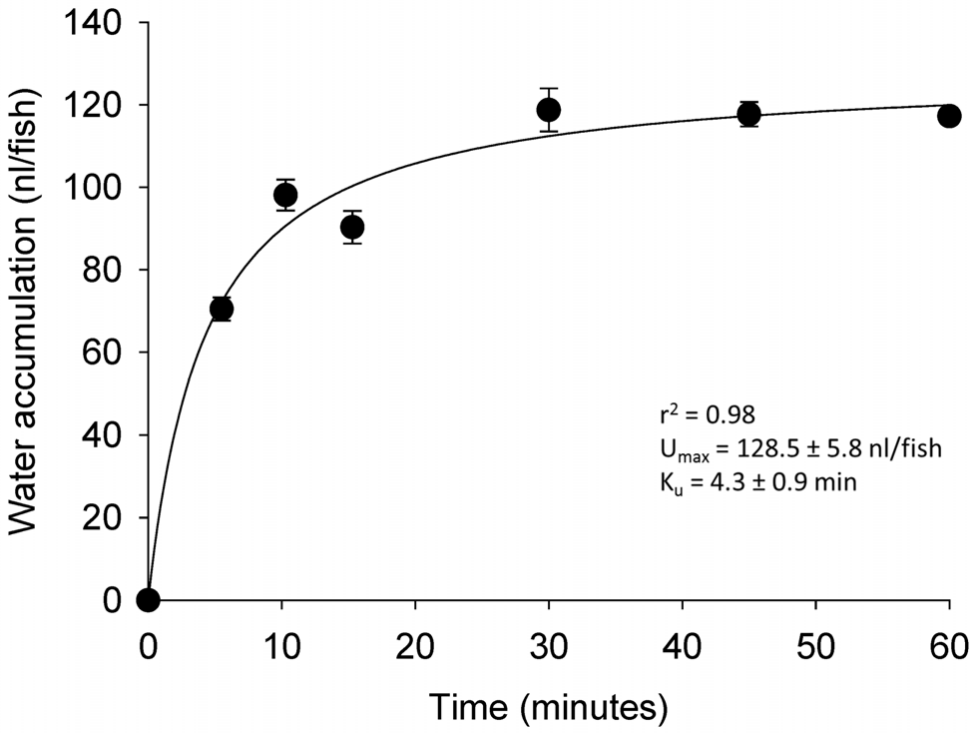
Characterization of water influx kinetics in larval zebrafish. The time-dependent accumulation of water in larval zebrafish at 4 dpf. The data were plotted using a hyperbolic equation: J_in_  =  (U_max_ x t)/(K_u_ + t); U_max_, maximum accumulation of water; t, exposure time (min); K_u_, half-time for saturation of water. Values are means ± SEM (*n* = 6).

The accumulation of water (^3^H_2_O) in zebrafish larvae increased with exposure time (Figure 1). The maximum accumulation and the half-time for saturation of water were 128.5±5.8 nl/fish and 4.3±0.9 min, respectively. The influx of 70 kDa dextran (a marker of drinking) appeared to be linear between 0 to 1.5 h (r^2^ = 0.94). The drinking rate was estimated to be 10.1±0.6 nl/fish/h, which accounted for <5% of total water influx.

### Water influx is reduced by phloretin

**Figure 2:**
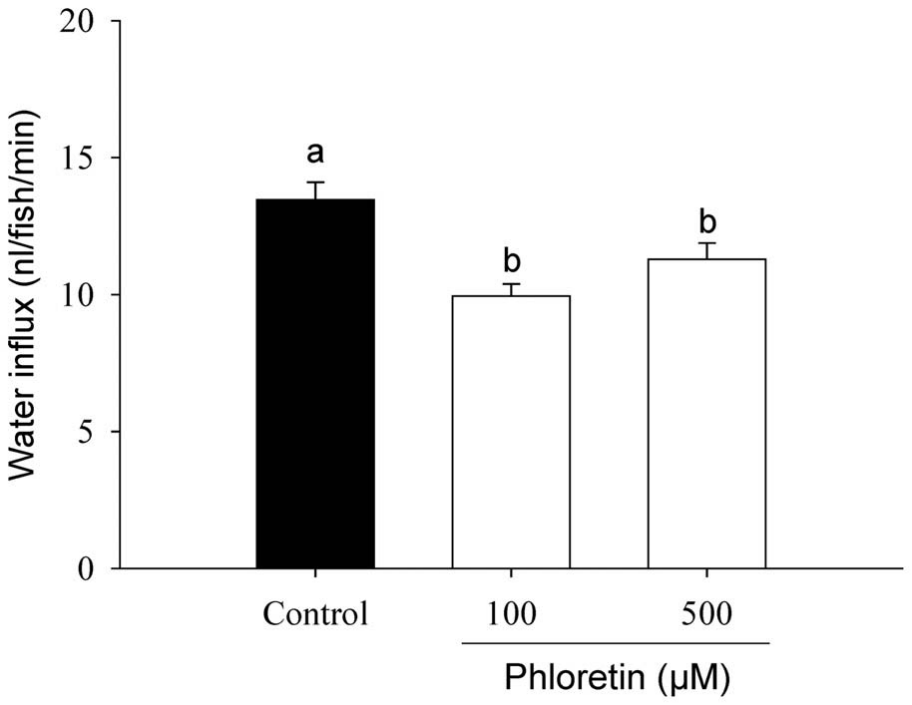
Phloretin reduces water influx in larval zebrafish. The effects of the putative aquaporin (AQP) inhibitor phloretin on water influx in larval zebrafish at 4 dpf. Values are means ± SEM (*n* = 6). Bars labelled with different letters represent a statistical difference (one-way ANOVA, followed by a post-hoc Holm-Sidak test; *p*<0.05).

The effects of the putative AQP inhibitors phloretin and acetazolamide on water influx were investigated. Exposure to 100 or 500 μM phloretin significantly reduced water influx (Figure 2), whereas acetazolamide (200 µM) was without effect (data not shown).

### Aquaporin-1a1 is expressed in ionocytes on the skin of the yolk sac

**Figure 3:**
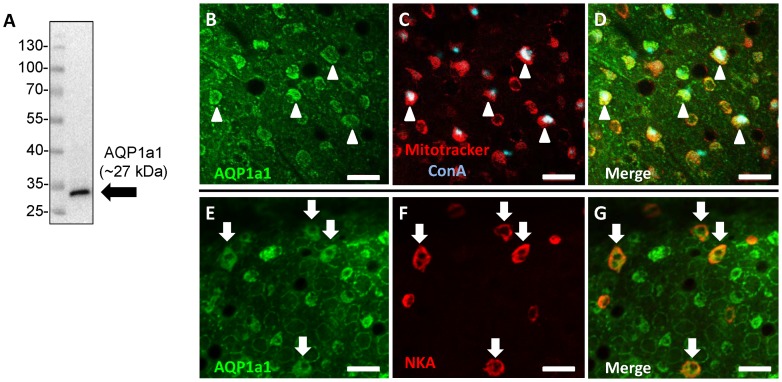
Aquaporin-1a1 is expressed in ionocytes on the skin of yolk sac in larval zebrafish. (A) A representative western blot showing that the eel aquaporin-1a1 (AQP1a1) antibody yielded a single immunoreactive band at ∼27 kDa in lysates of 4 dpf zebrafish larvae. (B) Fluorescent immunohistochemistry and confocal microscopy revealed that the expression of AQP1a1 on the skin of yolk sac (green), and a subset of cells stained positively with Mitotracker^®^ (red) and concanavalin-A (conA; blue). (D) is the merged images of B and C. Cells labelled with arrowheads represent areas of colocalization of AQP1a1 and Mitotracker^®^ or conA. (E) Some AQP1a1-positive cells (green) were also colocalized with (F) Na^+^/K^+^-ATPase staining (NKA; red). (G) is the merged images of E and F. Cells labelled with arrows represent areas of colocalization of AQP1a1 and NKA. Scale bar  = 25 μm.

Western blot analysis showed that the eel AQP1a1 antibody detected a single band at ∼27 kDa in lysates of 4 dpf larvae (Figure 3A). Immunohistochemistry and confocal microscopy revealed that AQP1a1 was expressed on the skin of the yolk sac at 4 dpf (Figure 3B). A substantial number of Mitotracker^®^- and ConA-positive cells were found to express AQP1a1 (Figure 3B, C and D). A subset of AQP1a1-positive cells was also found to colocalize with Na^+^/K^+^-ATPase staining (Figure 3E, F and G).

### Aquaporin-1a1 is predominantly expressed on the basolateral membrane of ionocytes

**Figure 4:**
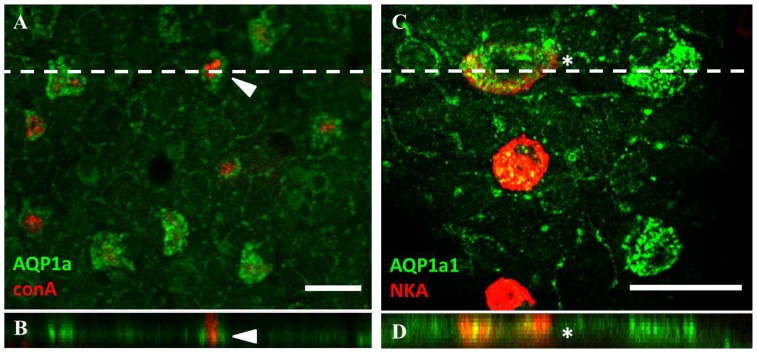
Aquaporin-1a1 is expressed predominantly on the basolateral membrane of ionocytes. Fluorescent immunohistochemistry and *z*-stack analysis with confocal microscopy revealed that (A and B) the expression of aquaporin-1a1 (AQP1a1; green) does not overlap with concanavalin-A staining (arrow; red). (C and D) However, AQP1a1 expression is found to co-localize with Na^+^/K^+^-ATPase (NKA; red) staining (cell labelled with an asterisk). Dashed lines in A and C indicate the position for *z*-stack images in B and D, respectively. Scale bar  = 20 μm.

To determine the cellular localization of AQP1a1 in ionocytes, *z*-stack confocal microscopy with ConA and Na^+^/K^+^-ATPase staining was performed (ConA and Na^+^/K^+^-ATPase stain the apical and basolateral membranes, respectively). Figure 4A shows immunostaining of AQP1a1 in ConA-positive cells. *Z*-stack analysis revealed that AQP1a1 did not overlap with ConA staining (Figure 4B). Figure 4C shows AQP1a1 staining in a subset of Na^+^/K^+^-ATPase-positive cells. *Z*-stack analysis suggested that AQP1a1 was co-localised with Na^+^/K^+^-ATPase staining at the basolateral membrane (Figure 4C).

### The protein expression of AQP1a1 is reduced by morpholino knockdown

**Figure 5:**
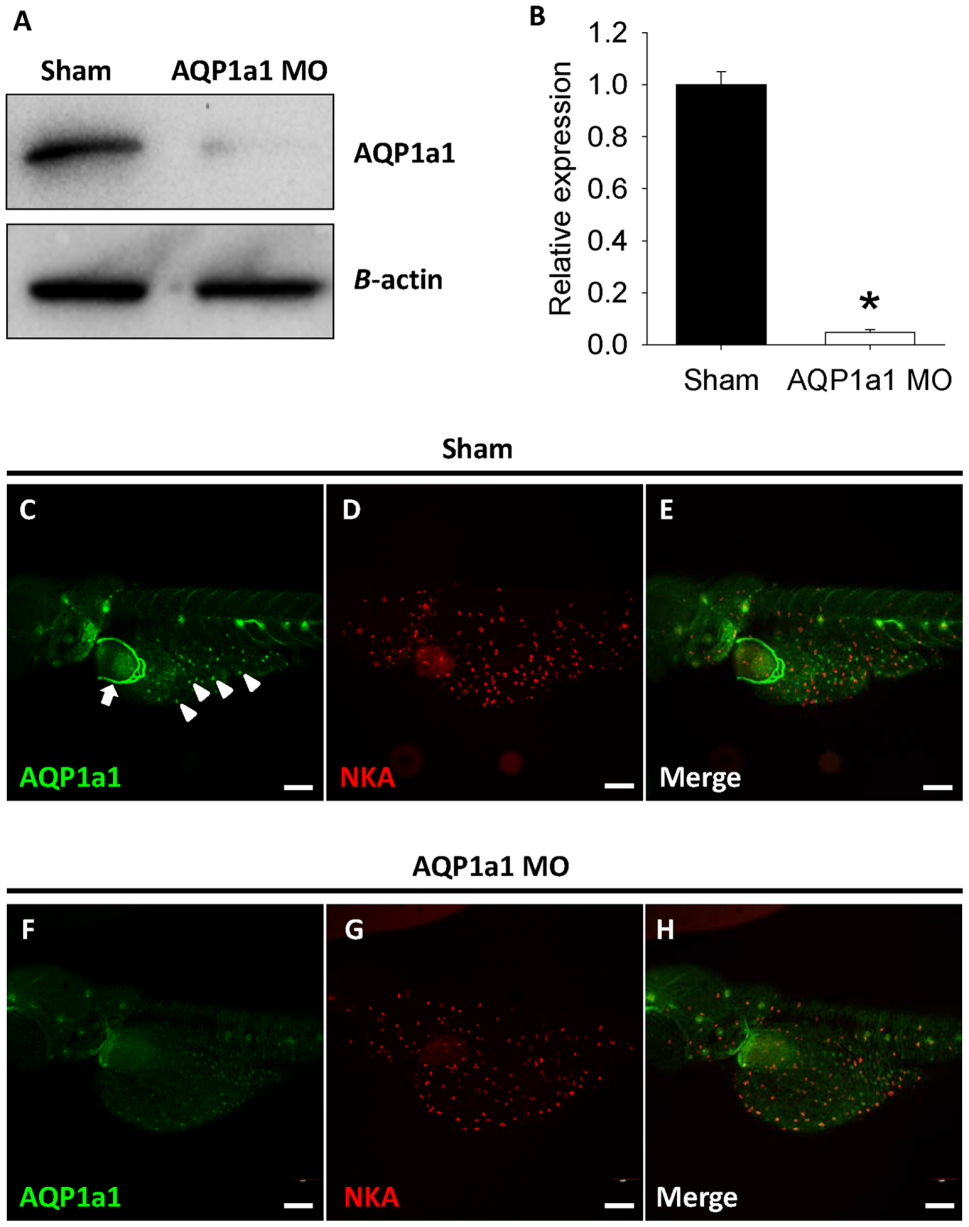
The protein expression of aquaporin-1a1 is reduced by morpholino knockdown. (A) A representative western blot showing the protein expression of aquaporin-1a1 (AQP1a1) in lysates of 4 dpf shams and AQP1a1 morphants (AQP1a1 MO) is reduced following morpholino knockdown. (B) Subsequent quantitative analysis revealed that the protein expression level of AQP1a1 in the morphants was significantly than shams (Student's *t*-test; *p*<0.05). Values are means ± SEM (*n* = 4). Fluorescent immunohistochemistry and confocal microscopy revealed the expression of (C) AQP1a1 (green; arrowhead) and (D) Na^+^/K^+^-ATPase (NKA; red) on the skin of yolk sac in the shams. AQP1a1 was also expressed in the red blood cells (Figure C; arrow). Figure (E) is a merged image of (C) and (D). (F) The expression of AQP1a1 was virtually absent in the morphants following the AQP1a1 knockdown. Figure (G) shows the expression of NKA, and Figure (H) is a merged images of (F) and (G). Scale bar  = 50 μm.

To determine the effectiveness of the morpholino knockdown on AQP1a1 protein expression, western blot analysis and immunohistochemistry were performed in shams and AQP1a1 morphants. As revealed by western blotting, the protein expression of AQP1a1 at 4 dpf was reduced following morpholino injection (Figure 5A). The expression of a housekeeping gene *β*-actin remained relatively constant after the morpholino knockdown. Subsequent quantitative analysis demonstrated that AQP1a1 protein expression was significantly reduced following the morpholino knockdown (Figure 5B). Immunohistochemistry showed that in shams, the AQP1a1 protein was expressed on the skin of yolk sac, as well as in red blood cells (Figure 5C). Figure 5D and 5E show immunostaining of Na^+^/K^+^-ATPase and the merged images of Figure 5C and 5D, respectively. In fish experiencing AQP1a1 knockdown, AQP1a1 expression was significantly lower when compared to shams (Figure 5F). Immunostaining of Na^+^/K^+^-ATPase was not affected by AQP1a1 knockdown (Figure 5G). Figure 5H shows the merged images of Figure 5F and 5G.

### Water influx is reduced by AQP1a1 knockdown

**Figure 6:**
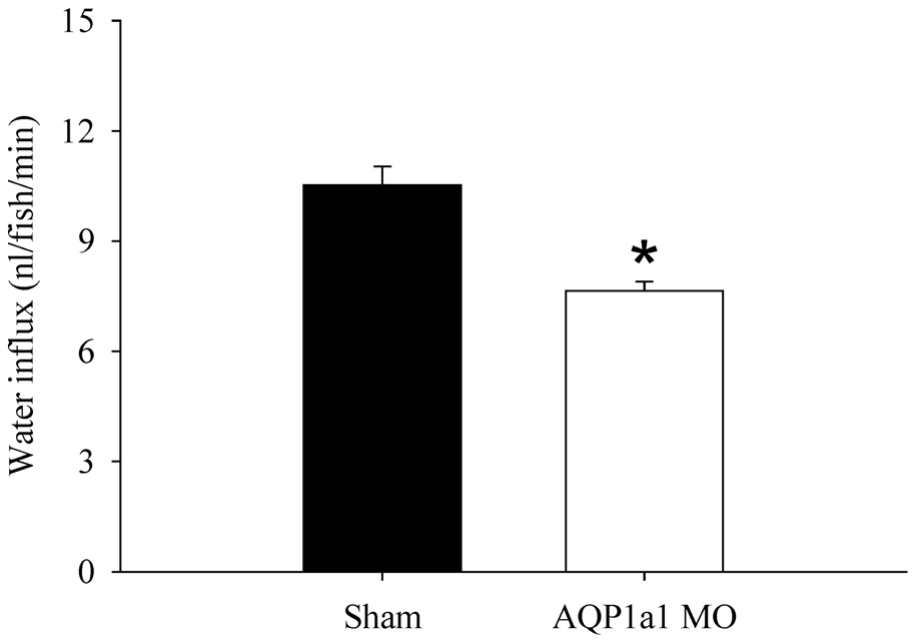
Morpholino knockdown of aquaporin-1a1 reduces water influx. The effects of aquaporin-1a1 (AQP1a1) knockdown on water influx in shams and AQP1a1 morphants (AQP1a1 MO) at 4 dpf. An asterisk indicates a significant difference between shams and morphants (Student's *t*-test; *p*<0.05). Values are means ± SEM (*n* = 6).

Knockdown of AQP1a1 significantly reduced water influx by about 30% in 4 dpf larval zebrafish (Figure 6). Using the paracellular permeability marker polyethylene glycol-4000 (PEG-4000), we observed that knockdown of AQP1a1 did not affect the paracellular properties (i.e. the absorption of PEG-4000 in the shams and AQP1a1 morphants was 3.3±0.2 and 3.0±0.4 fmol/fish/h, respectively; *p*>0.05; data not shown).

### Phloretin treatment reduces water influx in aquaporin-1a1 morphants

**Figure 7:**
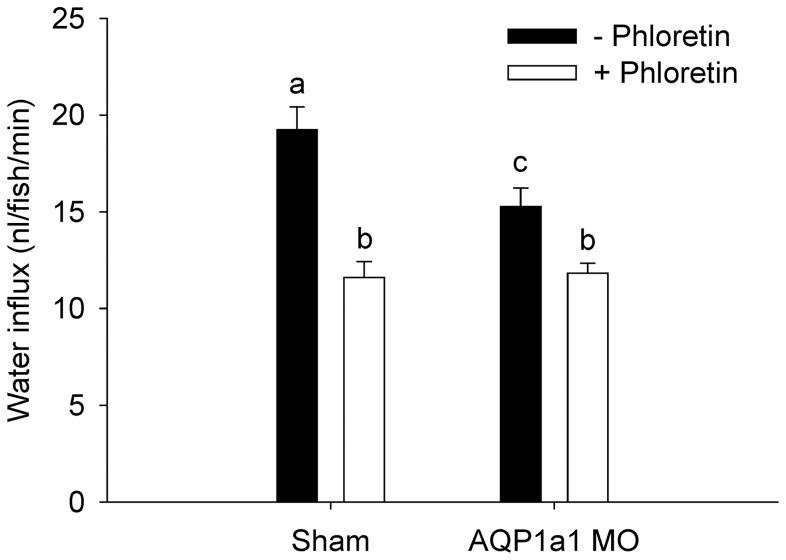
Phloretin treatment reduces water influx in aquaporin-1a1 morphants. The effects of the putative aquaporin (AQP) inhibitor phloretin on water influx in shams and AQP1a1 morphants at 4 dpf. Bars labelled with different letters represent a statistical difference (two-way ANOVA, followed by a post-hoc Holm-Sidak test; *p*<0.05). Values are means ± SEM (*n* = 6).

To evaluate whether other AQPs also contributed to water influx, water influx in 4 dpf AQP1a1 morphants was measured following phloretin treatment. The influx of water was significantly reduced after AQP1a1 knockdown or phloretin treatment (Figure 7). Water influx in the AQP1a1 morphants was also reduced significantly by phloretin treatment. Two-way ANOVA revealed a significant interaction between morpholino knockdown and phloretin treatment.

### Water influx is increased when the epithelial barrier functions are compromised

**Figure 8:**
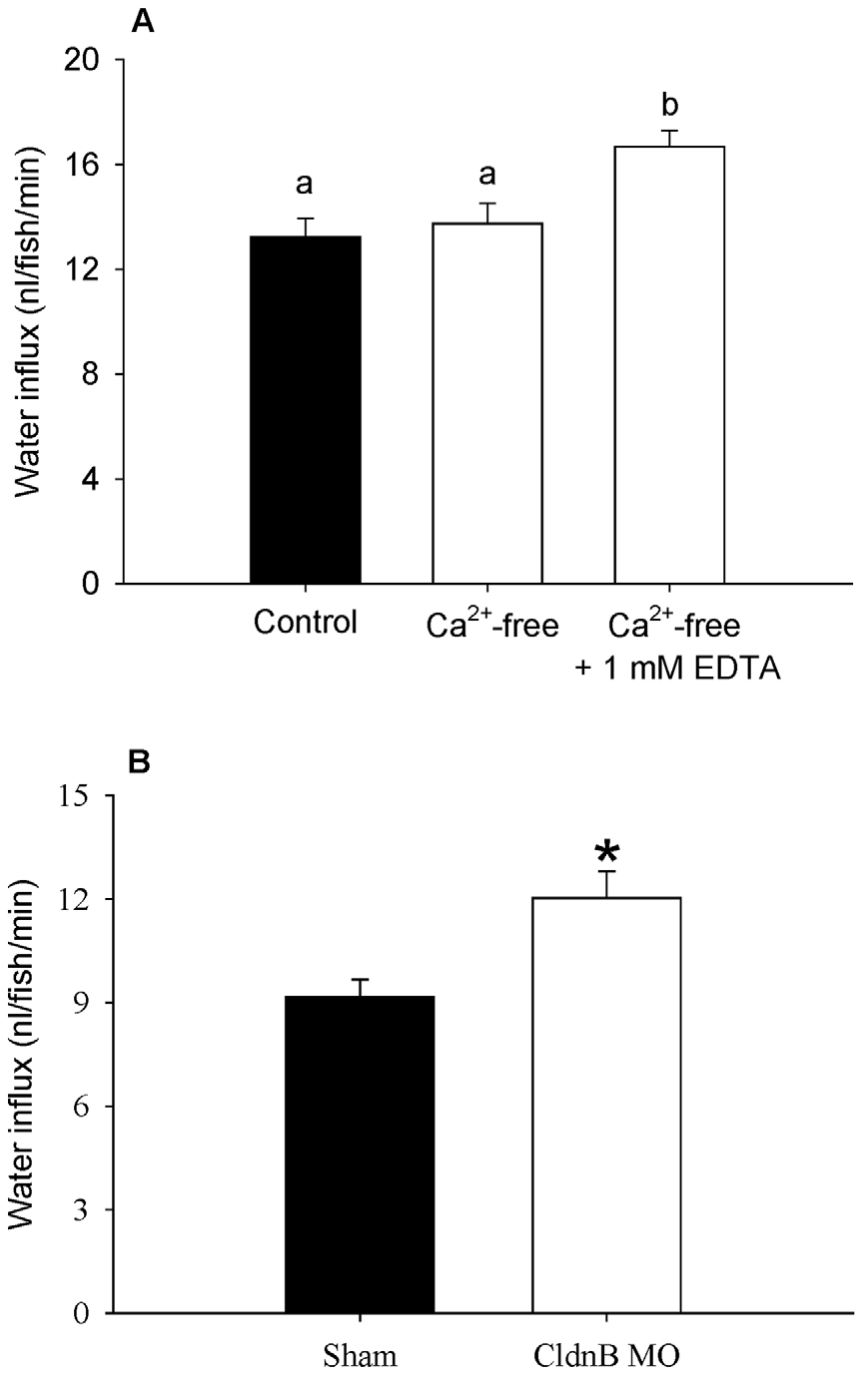
Evidence for the role of epithelial tight junctions in reducing paracellular water influx. (A) The influx of water in 4 dpf zebrafish larvae exposed to control, Ca^2+^-free and Ca^2+^-free plus 1 mM EDTA water. Bars labelled with different letters represent a statistical difference (one-way ANOVA, followed by a post-hoc Holm-Sidak test; *p*<0.05). Values are means ± SEM, *n* = 6. (B) The influx of water in shams and claudin-b morphants (CldnB MO) at 3 dpf. An asterisk indicates a significant difference between shams and morphants (Student's *t*-test; *p*<0.05). Values are means ± SEM, *n* = 6.

Fish exposed to 1 mM EDTA in Ca^2+^-free water exhibited a significant increase in water influx (Figure 8A). No statistical difference in water influx was observed between control (250 μM Ca^2+^ levels) and Ca^2+^-free water (in the absence of EDTA). Morpholino knockdown of claudin-b expression markedly increased water influx by about 25% in 3 dpf larvae (Figure 8B).

### Claudin-b protein expression is reduced in fish experiencing AQP1a1 knockdown

**Figure 9:**
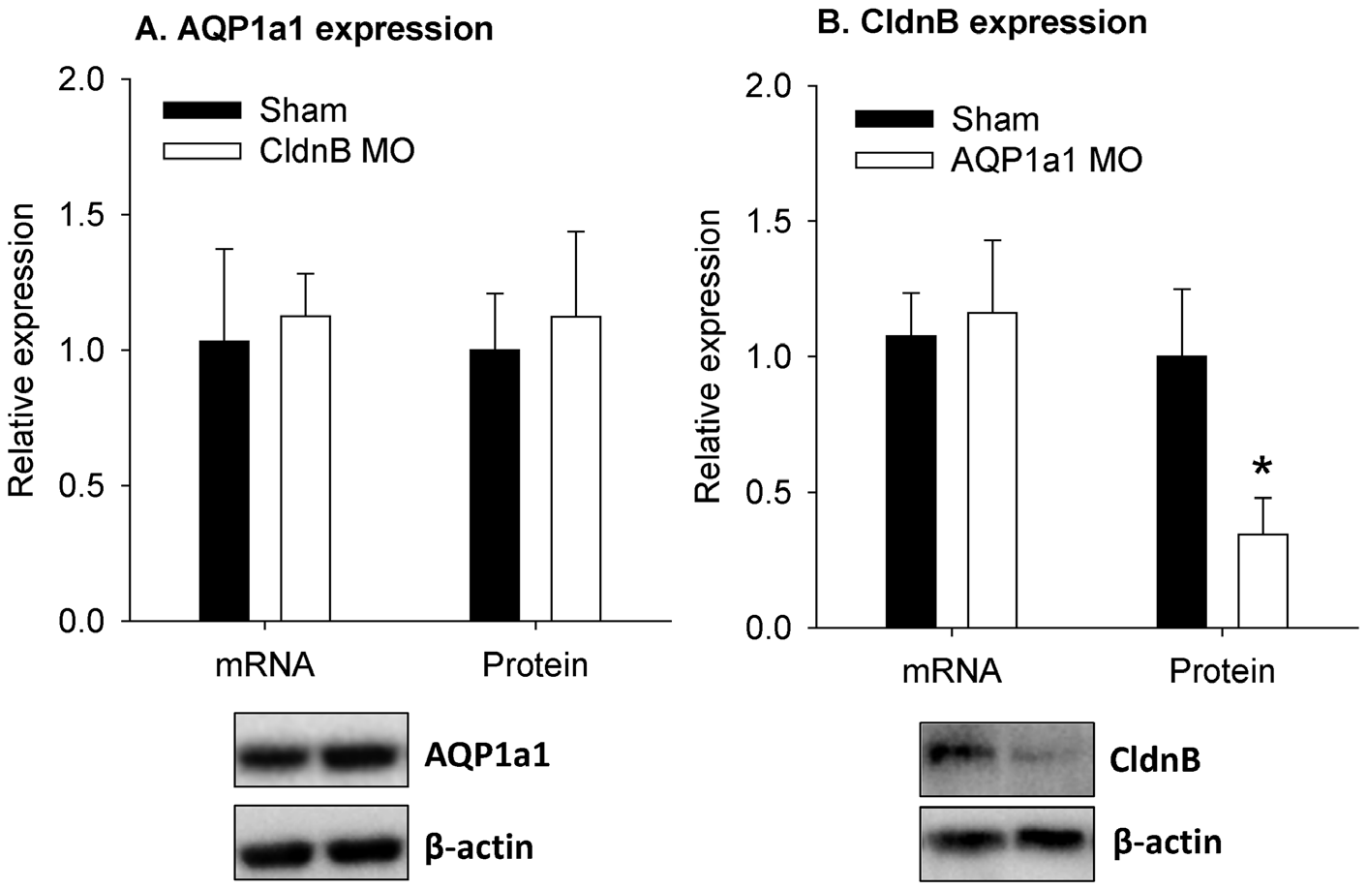
The protein expression of claudin-b is reduced in fish experiencing aquaporin-1a1 knockdown. The mRNA and protein expression levels of (A) aquaporin-1a1 (AQP1a1) and (B) claudin-b (CldnB) in shams and morphants experiencing CldnB or AQP1a1 knockdown. The mRNA and protein levels were determined by real-time PCR and western blot, using 18S and β-actin as the internal control, respectively. The representative western blots for AQP1a1 and CldnB are shown below each respective figure. An asterisk indicates a significant difference in protein level of CldnB between shams and AQP1a1 morphants (Student's *t*-test; *p*<0.05). Values are means ± SEM, *n* = 5–6.

Knockdown of claudin-b expression did not affect the mRNA or protein expression of AQP1a1 (Figure 9A). No change in the mRNA expression level of claudin-b was observed in AQP1a1 morphants; however, the protein level of claudin-b was significantly decreased in AQP1a1 morphants (Figure 9B).

## Discussion

### Overview

Although the general osmoregulatory strategies of teleost fishes inhabiting either FW or SW are well documented [1]–[3], the precise molecular mechanisms regulating water influx remain poorly understood. Recently, it was demonstrated that AQP1a1 is expressed on the skin of yolk sac in larval zebrafish [16], [17], and expression of zebrafish AQP1a1 in *Xenopus* oocytes was associated with an increase in water influx [7]. Here we show that in larval zebrafish, AQP1a1 is expressed predominantly on the basolateral membrane of ionocytes on the yolk sac epithelium. Furthermore, translational gene knockdown of AQ1a1 significantly reduces water influx, indicating its importance in facilitating transcellular water movement. Additionally, we provide evidence that epithelial TJs normally act to restrict paracellular water uptake in zebrafish larvae.

### Characterization of water influx

FW fish passively gain water and require its active removal by the kidney. In the present study, we observed that the half-time for saturation (K_u_) of water influx was 4.3 min, and appeared to reach steady state at 30 min. These findings imply that the turnover rate of water between the fish and the environment is high, and that the excretion of large volumes of water is required to maintain osmotic balance. Notably, it was previously demonstrated that glomerular filtration in zebrafish begins at about 2Using dextran dpf [33], [34]. The importance of the larval zebrafish kidney in fluid balance was demonstrated by severe edema formation in fish experiencing loss of pronephric kidney function [34]. In adult FW fish, the gills are the major site of water movement, accounting for about 90% of the total body water influx [3]. However, larval zebrafish do not possess a functional gill until 7Using dextran dpf [26], thus water influx before this developmental stage probably occurs either via drinking and/or directly through the skin. Using dextran (70 kDa) as a marker, we estimated that the drinking rate in 4Using dextran dpf larval zebrafish was about 10 nl/fish/h ( = 16.7 nl/mg dry weight/h). Because water influx was typically around 14Using dextran nl/fish/min (based on a 5-min flux period where the uptake rate remained linear; see Figure 1), it appeared that over 95% of water influx in 4 dpf zebrafish larvae occurred via the skin. This finding suggests that drinking is not an important component to overall water absorption in developing zebrafish.

### Water influx is reduced by phloretin treatment

Previous studies have shown that phloretin is an effective blocker of several AQP isoforms in mammals including AQP 1, 3, 7 and 9 [18]–[20]. The inhibitory mechanism of phloretin on AQPs is not well understood; however Huber et al. [35] have proposed that the inhibition of phloretin on AQPs is likely a result of electrostatic interactions between the ligand and amino acid residues within the water channel. In the present study, we also demonstrated that water influx was significantly reduced by phloretin, implicating AQPs in transcellular water transport. However, it is important to note that phloretin also inhibits urea transporters (UT); there is evidence both for and against a role for UT in facilitating water movement. Knepper et al. [36] showed that water and urea movement occurs through different pathways in the rat renal collecting tubules. Similarly, Martial et al. [37] reported that expression of mammalian UT-2 in *Xenopus* oocytes increased urea, but not water, permeability. However, Yang and Verkman [38] demonstrated that mammalian UT-3 can act as a water channel when expressed in *Xenopus* oocytes. It has also been suggested that mammalian glucose transporters (e.g., GLUTs) mediate water uptake when expressed in *Xenopus* oocytes, which can also be inhibited by phloretin [39], [40]. GLUTs are known to be expressed on the skin of larval zebrafish [41]; however, it is unclear whether teleost UT or GLUT orthologs can mediate water transport. It is to be noted though that, in contrast to the studies with *Xenopus* oocytes where both urea and water are moving in the same direction (from the external environment into the cells), the directions of net water and urea fluxes are in opposite directions across the skin of larval fish (urea is extruded into the external environment). Therefore, the influx of water through UT in larval zebrafish is expected to be low. In mammals, acetazolamide can inhibit water transport via AQPs [21], [22]; however acetazolamide did not affect water influx in the current study. The lack of an effect of acetazolamide on water influx was unexpected given the results of the phloretin experiment, but possibly due to difference in sensitivity to acetazolamide between mammalian and teleost AQPs. It has also been reported that mercury (Hg^2+^) at 10 mM can effectively reduce water fluxes *in vitro* via its inhibitory actions on AQPs [5], [7]. In the present study however, we observed that 10 mM Hg^2+^ caused significant mortality, and that 100 µM Hg^2+^ actually increased water influx by 30% (data not shown). Notably, Hg^2+^ is also known to adversely affect TJ integrity and increase paracellular permeability [42], [43]. The toxicity and the lack of specificity of Hg^2+^ prevent its use for evaluating AQP-mediated water transport *in vivo*.

### AQP1a1 is important in facilitating transcellular water influx

Recent studies using *in situ* hybridization demonstrated that AQP1a1 mRNA is expressed abundantly on the yolk sac of larval zebrafish [16], [17]. When expressed in *Xenopus* oocytes, AQP1a1 was found to induce water influx [7], providing direct functional evidence for AQP1a1 in transcellular water transport. In the present study, we observed that AQP1a1 protein was expressed on the skin of yolk sac. A substantial number of AQP1a1-positive cells were found to stain positively with Mitotracker^®^ and ConA. Additionally, a subset of AQP1a1-positive cells was enriched with Na^+^/K^+^-ATPase. These findings indicate that AQP1a1 protein is expressed in at least two sub-types of ionocytes, the H^+^-ATPase rich (HR) cell (identified using ConA or Mitotracker^®^), or the Na^+^/K^+^-ATPase rich (NaR) cell [44]. Interestingly, we also observed that AQP1a1 was expressed predominantly on the basolateral membrane in the ionocytes, implicating AQP1a1 as a possible regulator of ionocytes cell volume of and/or water movement into the blood circulation. In fact, translational gene knockdown of AQP1a1 reduced water influx by about 30%, suggesting that AQP1a1 plays a significant role in transcellular water uptake. Notably, phloretin treatment further reduced water influx in the AQP1a1 morphants by about 20%. Therefore, it is likely that additional pathways (e.g., other AQPs, UTs or GLUTs) at the apical membrane may also contribute to the overall transcellular water uptake. Additionally, we could not totally rule out the possibility that water influx occurred directly through the lipid bilayer, although its significance remains to be investigated. On the other hand, our results suggest that expression of the epithelial tight junction protein claudin-b was significantly reduced in fish experiencing AQP1a1 knockdown. Therefore, it is possible that water influx through paracellular routes was increased in the AQP1a1 morphants, although the overall water influx in the morphants was reduced.

In addition to its water transport function, AQP1a1 may also play a role in CO_2_ and NH_3_ excretion [16]. For example, it has been shown that expression of zebrafish AQP1a1 in *Xenopus* oocytes increased the permeability of CO_2_ and NH_3_ [16]. Similarly, co-injection of mammalian AQP1a and carbonic anhydrase cRNA into *Xenopus* oocytes increased CO_2_ permeation [45]. In humans, it has been suggested that AQP1 is a major pathway for CO_2_ flux across the erythrocyte membrane [46]. Therefore, it is possible that AQP1a1 may function as a gas channel to mediate CO_2_ and NH_3_ diffusion and indeed water transfer may be secondary to these functions. On the other hand, modulation of AQP expression in response to changes in environmental salinity is well documented in teleost fish [14], [47]–[50]. Using an AQP1-GFP fusion protein in HEK293 cells (human embryonic kidney cell line), Conner et al. [51] recently demonstrated that exposure to dilute environment causes internalization of AQP1 into the cytoplasm within 10Using dextran s. Therefore, if similar regulatory systems exist in fish, the expression of AQP1a1 on the basolateral membrane of ionocytes in larval zebrafish may potentially provide a rapid mechanism for fine-tuning the volume of ionocytes, and/or regulating water movement into the blood stream.

### A tight epithelium is required to prevent excessive paracellular water influx

Although it is generally believed that water may diffuse across epithelia via paracellular pathways [52], no direct functional evidence has yet to show any role for TJs in restricting paracellular water flux. Calcium is critical for the maintenance of cell-cell junctions, and removal of Ca^2+^ by EDTA is known to disrupt the integrity of TJs [31]. In the present study, we observed that exposure of fish to EDTA caused a pronounced increase in water influx. Moreover, knockdown of claudin-b, an epithelial junctional protein that promotes a tight epithelium in larval zebrafish [24], caused a significant increase in water influx. These findings indicate that TJs (at least those incorporating claudin-b) normally act to restrict paracellular water influx. In the gall bladder of *Necturus,* it was reported that a significant component of water movement occurs through paracellular routes [53]. In contrast, water uptake is suggested to occur predominantly via transcellular routes in the intestine of either FW- or SW-acclimated rainbow trout [54]. Similarly, water uptake was shown to occur primarily via transcellular pathways in the intestine of the euryhaline fish *Fundulus heteroclitus* [42]. In the present study, it is difficult to experimentally distinguish the relative partitioning of water movement between transcellular and paracellular pathways *in vivo*. However, considering that 30% of water influx occurs through AQP1a1, and that presumably other transporters and channels (other AQPs, UTs or GLUTs) may also contribute to the overall water influx, it seems reasonable to suggest that a significant proportion of water uptake occurs through transcellular routes. Water influx may potentially occur through paracellular routes as well; however, the contribution of water influx via the paracellular pathways under normal conditions remains to be determined. Nevertheless, results from our study suggest that such pathways may become significant when the general epithelial permeability is increased.

## Conclusions

The present study demonstrated that in larval zebrafish, the water channel AQP1a1 protein is expressed predominantly on the basolateral membrane in HR and NaR ionocytes where it is presumed to have a role in regulating the volume of ionocytes, and/or water movement into the blood circulation. Additionally, translational gene knockdown of AQP1a1 reduced water influx by about 30%, clearly indicating that AQP1a1 has a prominent role in facilitating transcellular water influx. On the other hand, water influx was significantly increased in fish following treatment with the Ca^2+^-chelator EDTA or knockdown of the epithelial tight junction protein claudin-b. These findings indicate that TJs normally act to restrict paracellular water influx. In humans, defects in AQPs have been associated with a variety of disorders such as failure of the kidney to properly reabsorb water [55], [56]. The results of the present study suggest that zebrafish may be a useful alternate model system to understand the pathophysiology of water balance disorders *in vivo*.
